# Chemistry, General, Medical, and Pharmaceutical

**Published:** 1873-09

**Authors:** 


*Chemistry; General, Medical and Pharmaceutical, including the Chemistry of the U. S. Pharmacopœia. A Manual of the General Principals of the Science and the application to Medicine and Pharmacy.* By John Attfield, Ph. D., F. C. S. Fifth edition. Revised from the Fourth (English) Edition by the Author. Philadelphia: H. C. Lea, 1873. Buffalo: T. Butler & Son.

The many readers and admirers of the former edition will be pleased to learn of the publication of a new edition of Prof. Attfield's work. The favor with which the previous edition was received, and the praises which it received from all has induced the author to prepare the present edition with especial reference to the American Profession.

The chemistry of the preparations and Materia Medica of the U. S. Pharmacopœia has been introduced into this edition, which is some seventy- pages larger than the former. The chapter on the General Principles of Chemical Philosophy has been enlarged and entirely revised. The work is so well known to the majority of Medical students that it requires no further notice from our hands of its general contents, and we have only to heartily recommend it to their careful study.

